# Detection of Low Pathogenic Avian Influenza Virus Subtype H10N7 in Poultry and Environmental Water Samples During a Clinical Outbreak in Commercial Free-Range Layers, Netherlands 2017

**DOI:** 10.3389/fvets.2020.00237

**Published:** 2020-05-05

**Authors:** Evelien A. Germeraad, Armin R. W. Elbers, Naomi D. de Bruijn, Rene Heutink, Wendy van Voorst, Renate Hakze-van der Honing, Saskia A. Bergervoet, Marc Y. Engelsma, Wim H. M. van der Poel, Nancy Beerens

**Affiliations:** ^1^Wageningen Bioveterinary Research, Department of Virology, Lelystad, Netherlands; ^2^Wageningen Bioveterinary Research, Department of Bacteriology and Epidemiology, Lelystad, Netherlands; ^3^Poultry Department, GD-Animal Health, Deventer, Netherlands

**Keywords:** LPAIV, water, environmental sampling, poultry, outbreak

## Abstract

Wild birds are the natural reservoir of the avian influenza virus (AIV) and may transmit AIV to poultry via direct contact or indirectly through the environment. In the Netherlands, a clinically suspected free-range layer flock was reported to the veterinary authorities by the farmer. Increased mortality, a decreased feed intake, and a drop in egg production were observed. Subsequently, an infection with low pathogenic avian influenza virus was detected. This study describes the diagnostic procedures used for detection and subtyping of the virus. In addition to routine diagnostics, the potential of two different environmental diagnostic methods was investigated for detecting AIV in surface water. AIV was first detected using rRT-PCR and isolated from tracheal and cloacal swabs collected from the hens. The virus was subtyped as H10N7. Antibodies against the virus were detected in 28 of the 31 sera tested. An intravenous pathogenicity index (IVPI) experiment was performed, but no clinical signs (IVPI = 0) were observed. Post-mortem examination and histology confirmed the AIV infection. Multiple water samples were collected longitudinally from the free-range area and waterway near the farm. Both environmental diagnostic methods allowed the detection of the H10N7 virus, demonstrating the potential of these methods in detection of AIV. The described methods could be a useful additional procedure for AIV surveillance in water-rich areas with large concentrations of wild birds or in areas around poultry farms. In addition, these methods could be used as a tool to test if the environment or free-range area is virus-free again, at the end of an AIV epidemic.

## Introduction

In recent decades, the avian influenza virus (AIV) has caused many infections in poultry worldwide, leading to significant economic and animal welfare implications (1, 2). Most AIVs are low pathogenic avian influenza viruses (LPAIVs), which cause no to mild clinical signs in poultry, such as respiratory symptoms, a drop in egg production and a slight increase in mortality rates (3). However, viruses of the H5 and H7 AIV subtypes can evolve to highly pathogenic avian influenza viruses (HPAIVs), which cause severe systematic infections and high mortality rates in poultry (4, 5). These severe clinical signs of these HPAIVs are the reason that prevention of AIV infections in poultry is required. If poultry is infected with H5 and H7 subtypes in spite of this, poultry must be culled, to prevent the spread of these viruses (6).

Poultry in free-range areas can come in contact with the virus through infected wild birds, especially aquatic birds from the order *Anseriformes* and *Charadriiformes*, which are the natural reservoir of AIV (7). Wild birds shed the virus via the respiratory and/or cloacal route and transmit the virus either directly by host-to-host contact, or indirectly through the environment (8). Once the virus is shed in the environment, it can remain infective for varying lengths of time depending on environmental variables such as water temperature and type (9), the number of micro-organisms present (10) and the amount of UV radiation by solar light (11). Because the virus is often shed in aquatic environments by wild birds, the detection of AIV in water is particularly relevant. Therefore, various methods have been developed for detecting AIV in water. Several studies have reported methods to detect AIV in surface water during field infections of AIV (12–15), however, most of these studies only showed the efficiency of their methods under laboratory circumstances (16–24).

In this study, we developed two methods that are able to detect AIV in natural water samples after field sampling. These methods can be useful screening tools to determine whether AIV is circulating in the environment. Such a tool has several applications. First of all, EU countries monitor the circulation of AIV in their environment using dead wild bird surveillance programs (25). In such a surveillance, dead found wild birds are examined for AIV: AIV positive dead wild birds indicate that the virus may be circulating in the area where the bird was found. Screening surface water for the presence of AIV could assist this surveillance (26) particularly when AIV surveillance fails due to HPAIVs that do not cause noticeable mortality in wild birds. The second use becomes apparent when HPAIV is already circulating: additional precautionary measures can then be taken, such as keeping free-range poultry indoors (Government of the Netherlands) (27). Screening of surface water for the absence of HPAIV can support the decision to lift the precautionary measures at the end of a HPAIV epidemic. Despite the putative advantages of screening for AIV in surface water, this is currently not performed in the Netherlands.

This manuscript describes diagnostic procedures and additional experimental research that was performed to confirm an infection with an LPAIV H10N7 virus in a free-range layer flock showing clinical signs. The infection on the farm provided an unique opportunity to test two environmental diagnostic methods that were developed, and their potential for detecting AIV in surface water. If this pilot study yields promising results, either or both of these methods could be used in the future for screening of AIV in the environment.

## Materials and Methods

### Diagnostics of Hens

#### Virus Detection

##### Real-time reverse transcriptase polymerase chain reaction (rRT-PCR)

Tracheal and cloacal swabs were taken from 21 hens which were lethargic and had a loss of body condition, which were the clinical signs representative for the flock. At the laboratory, the dry swabs were pooled in 10 ml 2.95% Tryptose phosphate and 1% Gentamicin. A pool consisted of five swabs of the same swab type (tracheal or cloacal). RNA was extracted from the pools using the MagNA Pure 96 System (Roche, Almere, Netherlands) and the MagNA Pure 96 DNA and Viral NA Small Volume kit (Roche) following manufacturer's instructions. Total RNA was tested by rRT-PCR targeting the influenza M gene (M-PCR), which detects all AIV subtypes, as described previously (28). In addition, the sequence of the haemagglutinin (HA) cleavage site and the neuraminidase subtype was determined by Sanger sequencing in two cloacal swab pools (29).

After sequencing, the isolated RNA was tested in an H10-specific rRT-PCR. The sequences of the primers (RF5127 5′-TGTGCATCGCATGTTTCCAT-3′ and RF5133 5′-CATCATTCTCTGGTTTAGCTTCGG-3′) and probe (RF5134 5′6-FAM-TGACAACGGCTAGAAGAACAAAACATGATGCC-BHQ-3′) were kindly provided by Ron Fouchier, Erasmus MC, Netherlands. One-step rRT-PCR was accomplished with a MX3005p cycler (Agilent, Amstelveen, Netherlands) by use of the OneStep RT-PCR Kit (Qiagen, Venlo, Netherlands). The manufacturer's instructions of the kit were followed with 1.25 mM MgCl_2_, 0.3 μM Rox, 0.6 μM of each primers, and 0.1 μM of the probe added to the mix. Finally, 5 μl of RNA was amplified in a volume of 25 μl by employing the following temperature profile: 50°C for 30 min and 95°C for 15 min, followed by 45 cycles of 94°C for 10 sec, 58°C for 30 sec, 72°C for 10 sec.

##### Virus isolation

A tracheal and a cloacal swab pool were each inoculated into the allantoic cavity of four 9- to 11-day-old specific pathogenic free embryonated chicken eggs (0.2 ml/egg) following the guidelines of the OIE (30). The eggs were incubated for up to 6 days at 37°C. After 6 days, or earlier when the embryo's died, the eggs were chilled at 4°C for 4 h or overnight and the allantoic fluid was harvested. The allantoic fluid was tested for the presence of haemagglutinating activity and the absence of bacterial contamination. Fluids giving a negative haemagglutination reaction were passed into another four eggs.

#### Serology

Two days after virus detection, blood samples were collected from 31 randomly selected hens at the farm. At the laboratory, blood was centrifuged (20 min at 1780g) to obtain sera, which were subsequently inactivated in a water bath of 56°C for 30 min. Sera were tested for the presence of antibodies against AIV using the nucleoprotein-blocking ELISA and the Haemagglutination Inhibition (HI) assay.

##### ELISA

The employed indirect double-antibody sandwich ELISA detects antibodies against all AIV subtypes and is a modified version of the nucleoprotein-blocking ELISA described previously (31). The antigen, an H7N1 virus treated with 1% Nonidet P40, binds to the monoclonal nucleoprotein HB65 (Wageningen Bioveterinary Research, Lelystad, Netherlands) that is used to coat the ELISA plates. Serum was diluted (1:8) with an ELISA buffer (1% Difco™ Skim Milk and 0.05% Tween 20) and was added to the antigen. If present, antibodies were bound to the antigen and were marked by the horseradish peroxidase conjugate.

##### HI assay

The used HI assay is a classic laboratory procedure for the subtyping of antibodies of AIV and is based on the inhibition of the agglutination reaction by haemagglutinin subtype-specific antisera (3). Auto-agglutination was avoided by incubating the sera with chicken erythrocytes as described previously (32). The HI assay was performed according to the methods described in the OIE manual using eight haemagglutination units of virus instead of four (30). Sera were tested against the antigens of subtype H10N4 and H10N9. HI assay results were reported as log_2_ HI titres, with titres ≥3 log_2_ considered positive.

#### Assessment of AIV Virulence: Intravenous Pathogenicity Index (IVPI)

The IVPI was determined for the virus (A/chicken/Netherlands/17013178-006010) that was obtained from the cloacal swabs. The IVPI was performed according to the method described in the “Diagnostic Manual for avian influenza” (33). Ten 6-week old specific-pathogen-free layers were inoculated intravenously with the virus, after which the layers were monitored for clinical signs and mortality for 10 days. The experiment was approved by the Central Animal Experiments Committee (license number 1631018100).

#### Pathological Examination

##### Pathology

Four hens, including one dead hen and three hens with clinical signs representative for the clinical situation in the flock, were submitted to GD Animal Health, Deventer, the Netherlands, for a general pathological examination. When arrived, the three living birds were stunned by exposure to a mixture of carbon dioxide and oxygen and then exsanguinated. Routine necropsy procedures were performed, including parasitological, and histological examination. In addition to the necropsy procedures, PCRs were executed to exclude infections with other pathogens or co-infections. Therefore, samples of the trachea were tested for the presence of Infectious bronchitis virus (34), *Mycoplasma gallisepticum* (35) and *Mycoplasma synoviae* (36). Standard PCR methods used by GD Animal Health excluded tracheal infections with Infectious laryngotracheitis virus and Avian metapneumovirus, and oviduct infections with Group I Aviadenovirus and Atadenovirus (Egg drop syndrome virus). Furthermore, immunohistochemical staining excluded infections with Ornithobacterium rhinotracheale and Chlamydia psittaci in the air sacs (data not shown).

##### Histology

Samples of trachea, lung, air sac, duodenum and shell gland were fixed in 4% neutral buffered formalin, embedded in paraffin, sectioned at 2 μm, and stained with hematoxylin and eosin (H&E) for light microscopic examination. In the same organs, the presence of influenza A virus antigen was investigated using immunohistochemistry (IHC). For IHC, samples were fixated for at least 24 h in buffered 10% formalin, followed by dehydration in absolute ethanol and embedding in paraffin wax, sections were cut at 4 μm and mounted on glass slides. Endogenous peroxidase activity was blocked by incubation with 1% H_2_O_2_ containing 0.1% NaN_3_ for 20 min at room temperature (RT) and subsequently boiled in Tris (0.01 M) EDTA (0.001 M), pH 9.0 for 10 min. The binding of Fc-receptors was blocked by incubation with 10% fetal bovine serum for 20 min at RT. The immunostaining of influenza A virus-positive-cells was performed using 1:1,000 diluted anti-influenza A virus nucleoprotein monoclonal antibody (Meridian Life Science, Memphis, USA) in Normal Antibody Diluent (Klinipath, Duiven, Netherlands) for 30 min at RT. After three subsequent wash steps with phosphate-buffered saline, the sections were treated with anti-mouse Dako EnVision+ (Dako UK Ltd, Cambridgeshire, UK) for 30 min at RT. Again, sections were washed three times with phosphate-buffered saline and then treated with DAB+ (Dako UK Ltd) for 5 min at RT. Finally, the sections were counter-stained using haematoxylin. Sections incubated in the absence of primary antibody were taken along as negative controls.

### Diagnostics of Water Samples

#### Water Sampling

**Figure 1:**
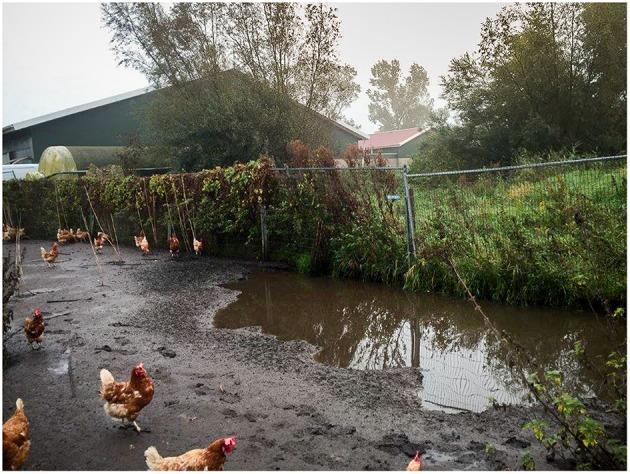
Puddles were formed in the free-range area after severe and prolonged rainfall. Water samples were collected from these puddles.

**Figure 2:**
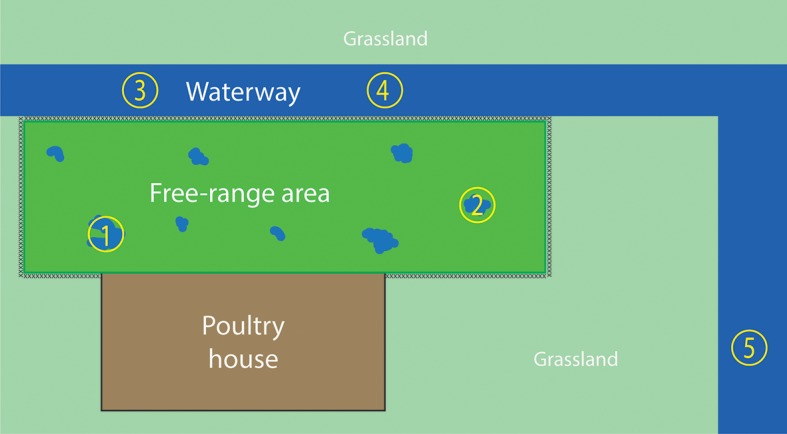
Overview of the water sampling sites. The fenced free-range area is enclosed by the poultry house and waterway. Water samples 1 and 2 were collected from the puddles of water in the free-range area, which were caused by severe rainfall. Water samples 3, 4, and 5 were collected from the waterway. Sampling sites 1–4 were sampled during both farm visits; sampling site 5 was sampled only during the second visit.

Water samples were collected from the free-range area and a waterway around the farm, 2 days after the virus detection in hens. The free-range area is a fenced grassland connected to the poultry house that allows the hens going outside during daylight. After severe and prolonged rainfall, puddles of water were formed in the free-range area (Figure 1). During the first visit, two water samples were collected from the puddles of water in the free-range area, and two water samples were collected from the waterway (Figure 2). The samples were collected with a bucket attached to a stick to avoid disturbing the sampling sites and avoid cross-contamination between the sampling sites. Samples were taken in the middle of the water puddle and at least 1 meter from the ditch side of the waterway. Within each sampling site, a 1 liter sample and a 50 liter sample of water were collected.

After the virus was detected in hens, they were prohibited to enter the free-range area any longer. Because the hens could not excrete new virus in the free-range area, there was a possibility to examine the survival of the virus in the environment. Therefore, the farm was visited 2 weeks after the first water sampling moment. During the second visit, 14 days after the first visit, the sampling sites of the first visit were sampled again plus an additional site of the waterway (sample site number 5 in Figure 2).

#### Detection of AIV in 1 Liter Water Samples: “The 1L Method”

Water samples were stored at 4°C before being filtered within 12 h of sampling. One liter of water was divided into two conical centrifuge tubes (Corning® 500 mL PP Centrifuge Tubes, Thermofisher, Breda, Netherlands) and centrifuged in a Mistral 6000 (MSE, Heathfield, UK) for 15 min at 1781 g at 4°C. After centrifuging, the supernatant was filtrated using a Sterifil™ 47 mm Aseptic Vacuum Filter System and Holder (Millipore, Merck, Darmstadt, Germany) and two different types of filters. The first filter, a hydrophilic Glass Fiber Filter with a pore size of 1.0 μm without binder resins (Millipore, Merck), was used for liquid clarification and was replaced when the filter was constipated. The second filter, a hydrophilic mixed cellulose esters membrane (MF-Millipore™ Membrane Filter, Merck) with a pore size of 5.0 μm, is able to catch the virus if present. After processing the sample, the membrane was collected from the filter system into a bead tube and frozen (−80°C) until the start of the RNA isolation. In addition, the filter system was cleaned by soaking the system in a chlorine solution (Suma Tb D4, Diversey, Utrecht, Netherlands)for 10 min, followed by rinsing the system with demi water.

RNA was isolated from the membrane with the PowerWater® RNA Isolation kit (Qiagen). Manufacturer's instructions were followed, with several modifications: (1) the prescribed vortex adapter was not used; instead the bead tubes were horizontally vortexed manually on the vortex (IKA® MS1 minishaker, Merck), (2) the fluid was centrifuged for 3 min at 3000 g instead of 1 min at 4000 g at point 9, and 3) the RNA was eluted in 50 μl RNase-free water to obtain a higher concentration of RNA. The eluate was frozen (-80°C) until it was processed in the M-PCR.

#### Detection of AIV in 50 Liter Water Samples: “The 50L Method”

Water samples were stored at 4°C before filtering for a maximum of 24 h. First, large substances, such as water plants, were filtered out of the water samples by flowing the water through a clean towel. Secondly, 200 μl of Porcine Epidemic Diarrhea virus (PEDV) with a titer of 6.3 × 10^4^ plaque-forming units/ml was added as an intern control. After these preparations, the water was filtered through a Rexeed™-25A hemodialyzer dead-end hollow fiber ultrafilter (Asahi Kasei Medical, Brussel, Belgium) using a peristaltic pump (Geopump™ series II, Geotech, Haarlem, Netherlands) and sterilized silicon hoses (drive 1, speed 2.5). After processing the sample, the filter system was rinsed with 0.5L of tap water to flush the last part of the sample from the silicon hoses into the filter. The filter was stored overnight at 4°C, when necessary. The virus was flushed from the filter during a 20-min back flush with 500 ml TGBE buffer [500 ml RNase-free water, 6.05 gram Tromethamine, 1.9 gram glycine, and 10 gram beef extract (Bacto™ Beef Extract Dessicated, Life Technologies, Thermofisher)]. After back flushing, the TGBE buffer containing virus was divided over two conical centrifuge tubes (Corning® 500 mL PP Centrifuge Tubes, ThermoFisher) and centrifuged for 2 h at 4500 g at 4°C. After centrifuging, the pH was set to a pH of 7 by adding 5M HCL and 125 ml 5x PEG/NaCl (500 gram PEG 8000 plus 87 gram NaCl and filled with distilled water up to 500 ml). After an incubation period of 1 h (or overnight) at 4°C, the centrifuge step was repeated and the supernatant was removed. To make the pellets more compact, the tubes were centrifuged for another 10 min at 4500 g at 4°C and again the supernatant was removed. Each pellet was resuspended in 500 μl Dulbecco's Modified Eagle Medium (Gibco, ThermoFisher). Finally, 500 μl of the fluid was added to 2 ml of NucliSens lysis buffer (bioMérieux, Boxtel, The Netherlands) and stored for maximum 24 h at 4°C.

RNA was isolated using the Nuclisens® Magnetic Extraction Reagents and the Nuclisens® MiniMAG® (bioMérieux) according to the manufacturer's instructions. The eluate was frozen (−80°C) until it was processed in the M-PCR.

### Full Genome Sequencing and Phylogenetic Analysis

Next-generation sequencing (NGS) was performed to determine the full genome sequences of the AIV isolated from the hen (EPI_ISL_394155) and from the 1L water sample that was collected at sampling site 1 during the first visit (EPI_ISL_394156). Illumina sequencing was performed as described previously (37).

For phylogenetic analysis, a dataset was composed of HA nucleotide sequences of the top 100 blast hits (on March 10, 2020) from the GISAID EpiFLu Database (https://www.gisaid.org), using the HA segment of A/Chicken/Netherlands/17013178-006-010/2017 as query. The collected sequences were aligned using Multiple Alignment with Fast Fourier Transformation [MAFFT v7.427; Katoh and Standley (38)]. Subsequently, a maximum likelihood tree was constructed by using RaxML version 8.2.12 (39) based on the general time-reversible (GTR) model of nucleotide substitutions with a gamma-distributed variaten of rates and 1,000 bootstrap replicates. The tree with the highest log likelihood was visualized using FigTree v1.4.4 (http://tree.bio.ed.ac.uk/software/~figtree).

## Results

### Outbreak Description

**Figure 3:**
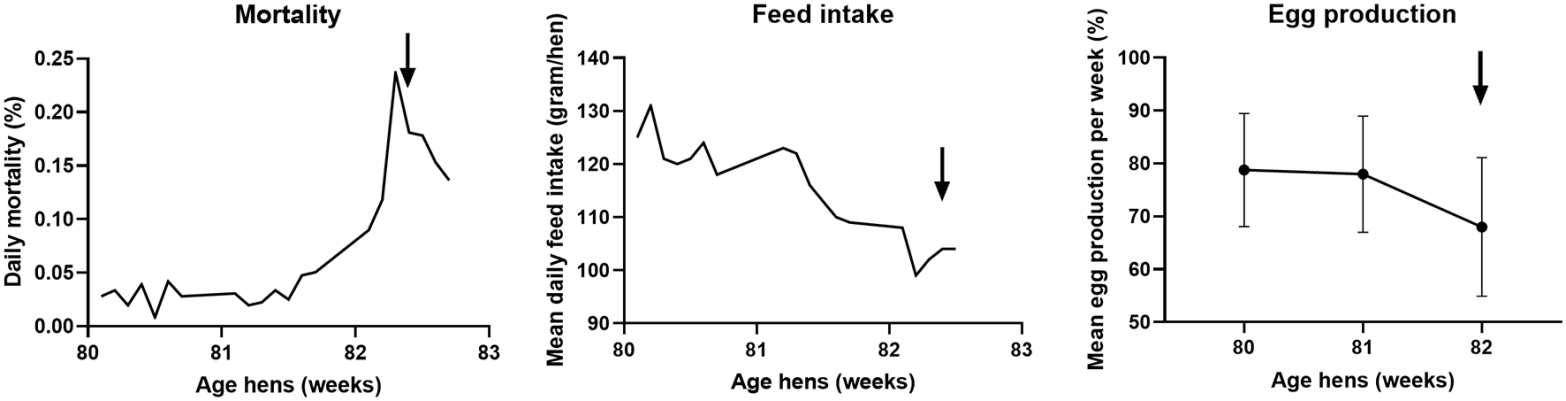
Course of daily mortality, mean feed intake and mean egg production of the flock between 80 and 83 weeks of age at the free-range layer farm. Daily mortality is expressed as percentage of the total amount of hens present, the mean daily feed intake in gram per day per hen, and the mean egg production per week as a percentage of the total amount of hens present ± standard deviation. The arrow indicates the day of reporting the clinically suspect situation.

In September 2017, the veterinary authorities received a report of clinical signs in hens of a commercial free-range layer farm. At the time of reporting, the farm housed 35,388 brown Novogen laying hens of 82 weeks of age in one house. The flock showed increased mortality, decreased feed intake and a drop in egg production as shown in Figure 3. The increased daily mortality, which started at 81 weeks of age, peaked with 0.24% between 82 and 83 weeks of age. During the mortality peak, mean daily feed intake decreased from approximately 120 gram per day to 100 gram per day per hen. The mean egg production per week decreased from about 78% at 80 and 81 weeks of age to 68% at 82 weeks of age. It is not likely that the virus was present in the flock for more than 8 weeks, because the flock had tested negative for antibodies against AIV during the national serological screening for AIV in poultry at 74 weeks of age (40).

Before reporting, the commercial free-range layer farm had experienced already four LPAIV introductions since the start of production in 2008. An H6N1 virus was detected in 2010 (41), followed by an H6N2 virus in 2012, an H9N2 virus in 2013, and an H6N2 virus in 2014. The farm is located in the northern part of the Netherlands, <2 km from the North Sea coast, and this area has plenty of waterways and lakes. This water-rich area provides a good habitat for wild (water) birds, which live in large quantities near the farm. Elbers and Gonzales (42) monitored the free-range area of this particularly farm with video-cameras for 1 year and reported various visits of wild birds to the free-range area (42). No direct contact between hens and wild birds was observed, and it is hypothesized that wild birds infected with AIV probably contaminate the environment—soil and water present in the free-range area—with AIV. Consequently, poultry may be infected through contact with contaminated soil or water in the free-range area. The type of farm, a free-range farm, together with the location of the farm, and therefore the presence of wild aquatic birds, may contribute to the multiple LPAIV introductions in the poultry farm. The presence of wild birds in the free-range area was also observed during the ifirst visit for water sampling: two mallards (*Anas plathyrhynchos)* were observed swimming in the puddle of water at sampling site 1 (Figure 2).

### Diagnosis of Hens

AIV was detected in all five tracheal and five cloacal swab pools using the M-PCR. The virus was subtyped as LPAIV H10N7 using Sanger Sequencing, and all swab pools tested positive in the H10-specific PCR. The virus was isolated by inoculation into specific pathogenic free embryonated chicken eggs and also subtyped as H10N7. The serum samples which were collected at the farm, tested positive for antibodies against AIV. The ELISA detected antibodies against AIV in 28 of the 31 sera. The HI assay detected H10 antibodies in 17 sera when tested against the H10N4 antigen, while 12 sera tested positive against the H10N9 antigen. The positive log_2_ HI titres ranged from 3 to 8. The pathogenicity of the isolated H10N7 virus was tested using an IVPI experiment. An IVPI score of 0.0 was measured. This score indicates that no clinical signs were observed during the experiment, which is in contrast to field observations.

#### Pathological Examination

##### Pathology

The hen that was submitted dead showed severe loss of body condition with loss of productivity. The ovary contained an ovarian adenocarcinoma with extensive peritoneal metastases. The euthanized hens presented a loss of body condition, characterized by muscular atrophy predominantly of the pectoral muscles. The head and appendices showed no abnormalities, the same for the lungs, but the tracheas were hyperemic. One hen had an extensive fibrinous airsacculitis, mainly of the thoracic air sacs. All hens had productive ovaries. Gastrointestinal contents were normal and despite a mild infection of small numbers of *Ascaridia galli* and *Heterakis gallinarum* no lesions were found. The liver, spleen and kidneys were normal. The abdominal fat pads were relatively normal in size. No other pathogens were detected by PCR in samples of the trachea and oviduct.

##### Histology

**Figure 4:**
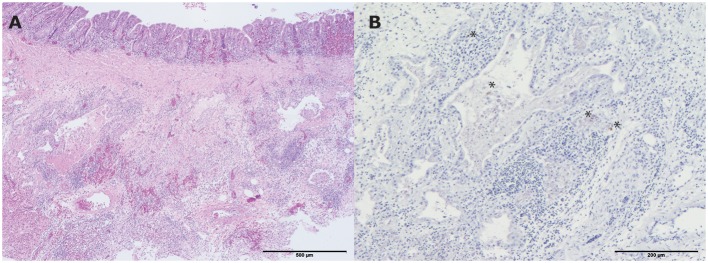
Histopathologic and immunohistochemical (IHC) testing results of a lung from one of the living hens that were submitted for pathological examination. **(A)** Mild pneumonia consisting of collapse of multiple atria and capillaries by moderate infiltrates of mainly macrophages, lymphocytes, less heterophils and fibrinous and necrotic debris with organization (hematoxylin and eosin (H&E) stain). Bar < 500 μm; **(B)** Influenza A virus antigen within the mononuclear infiltrate scattered cells. Bar < 200 μm. *indicates the colored cells.

All four hens showed a mild lymphoplasmacytic tracheitis, with intact epithelium and erythrocyte engorged capillaries (hyperemia). However, no viral antigen was demonstrated with IHC staining for influenza A virus in the tracheas. One of the four hens had a mild pneumonia, consisting of collapse of multiple atria and capillaries by moderate infiltrates of mainly macrophages, lymphocytes, less heterophils and fibrinous and necrotic debris with organization (Figure 4A). In addition, the epithelium of the parabronchi of this hen was hypertrophic and hyperplastic, while the lumen contained protein rich fibrillar debris (fibrin) with heterophils and macrophages. Although no viral antigen was present in epithelial cells or endothelial cells of this hen, the mononuclear infiltrate scattered cells contained viral antigen by IHC staining for influenza A virus (Figure 4B). Furthermore, this affected hen had a lymphohistiocytic airsaculitis with protein rich edema and accumulation with intraluminal protein rich debris and many large macrophages with foamy cytoplasm (active macrophages with signs of phagocytosis of debris). No viral antigen could be demonstrated by IHC staining for influenza A virus in the airsac.

The duodenum of all investigated hens showed mild villus atrophy and a moderate lymphoplasmacytic infiltrate with scattered heterophils within the lamina propria. The epithelium was intact, but numbers of intraepithelial lymphocytes were multifocally increased. Crypts appeared normal. No Influenza A viral antigen was demonstrated by IHC. The shell gland of two hens did not show any abnormalities, while in one hen that arrived alive scattered perivascular lymphocytic infiltrates were present in het deep stromal region of the secondary folds. The glandular and surface epithelium were not altered and Influenza A viral antigen could not be demonstrated by IHC staining.

### Diagnosis of Water Samples

Water samples collected on both sampling days from the free-range area and waterway were examined for the presence of AIV. The results of the M-PCR and H10-specific rRT-PCR for both methods are shown in Table 1. From the samples collected during the first sampling day, sampling site 1 resulted in positive H10-specific rRT-PCR results for both methods. It was sampling site 2—the other puddle in the free-range area—that was positive for both PCRs in both methods. No AIV was detected in the waterway samples. On day 14, the second sampling day, AIV was detected at sampling site 1 by both methods and PCRs. At sampling site 2, AIV was detected by the 1L method in both PCRs and by the 50L method only in the H10-specific rRT-PCR. The positive results of the second sampling day indicated that the virus is still detectable for at least 2 weeks. Again, no virus was detected in the samples collected from the waterway.

**Table 1:** Detection of AIV by the M-PCR and H10-specific rRT-PCR in water samples using the 1L and 50L water sample method.

**Water sampling site**	**Sample day 1**^**a**^				**Sample day 14**			
	**1L method**		**50L method**		**1L method**		**50L method**	
	**M-PCR**	**H10 PCR**	**M-PCR**	**H10 PCR**	**M-PCR**	**H10 PCR**	**M-PCR**	**H10 PCR**
1	No ct	35.27	No ct	35.21	35.19	34.94	35.16	35.67
2	31.87	30.49	29.06	29.91	33.86	33.29	No ct	36.11
3	No ct	No ct	No ct	No ct	No ct	No ct	No ct	No ct
4	No ct	No ct	No ct	No ct	No ct	No ct	No ct	No ct
5	NA	NA	NA	NA	No ct	No ct	No ct	No ct

a *Sample day 1 indicates the first moment water samples were taken. The farm was visited again two weeks after the first water sampling moment, sampling day 14, to examine the survival of the virus in the environment. This was possible because after virus detection the hens were prohibited to enter the free-range area any longer, and therefore no new virus could be excreted in the free-range area*.

*NA, not applicable, because no sample was taken. H10 PCR, H10-specific rRT-PCR*.

### Full Genome Sequencing and Phylogenetic Analysis

The full genome sequences of the viruses detected in the hens and in the water samples collected in the free-range area were determined by NGS. The genome sequences of these viruses were similar, and no specific mutations or adaptations which lead to any changes in amino acids sequences were identified. Therefore, it remains unclear whether the water in the free-range area was contaminated by infected wild birds, resulting in introduction of the virus into the poultry farm. Or, whether the free-range area was contaminated later, by the already infected chickens. Phylogenetic analysis of the HA gene segment was performed to study the relationship between the H10N7 virus detected at the farm and other poultry and wild bird viruses. The most closely related virus based on the HA gene segment was a H10N6 virus detected in a mallard in Denmark in 2011. No closely related H10N7 viruses were detected in poultry or wild birds in 2017 (Figure S1).

## Discussion

Post-mortem examination, diagnostics and additional experimental research confirmed an LPAIV H10N7 infection in a commercial free-range layer flock in the Netherlands. The circumstances surrounding this particular outbreak enabled us to investigate the potential of two methods for detecting AIV in the environmental water samples during a natural AIV infection. The described methods can be a useful additional procedure for AIV surveillance, or can be used as a tool to decide when precautionary measures, like keeping free-range chickens indoors, could be lifted at the end of an AIV epidemic. In contrast to the waterways near the farm, in which no AIV was detected, H10N7 virus was detected in the puddles of water in the free-range area. Both methods used for detecting AIV in water allowed the detection of H10N7 virus, demonstrating the potential of these diagnostic methods. Unfortunately, full genome sequencing did not provide conclusive insight into the epidemiology of the infection. Phylogenetic analysis did not reveal circulation of similar H10 viruses in wild birds or poultry in Europe in 2017.

The clinically suspect situation, as a result of the infection with the LPAIV H10N7 was observed and officially reported by the farmer. He observed an increase in mortality, a decrease in feed intake and a drop in egg production although none of these three parameters reached the official reporting thresholds described in the acting Statutory Regulation (43). These thresholds, originally implemented for the early detection of HPAIV, are set for laying flocks at ≥0.5% mortality per day for two consecutive days and ≥5% decrease in egg production or feed/water intake per day for two consecutive days. New effective thresholds for an early detection for HPAIV and LPAIV infections in poultry have recently been suggested (44). If those newly suggested thresholds would have been operational, the observed clinical signs would have led to mandatory reporting.

In contrast to the clinical signs observed on the farm, no clinical signs were observed during the IVPI experiment. There are several possible explanations for the observed difference between the field situation and the experiment. Firstly, the AIV infection in the field may have been accompanied by other viral or bacterial respiratory pathogens leading to more severe clinical signs. These kind of co-infections for AIV have previously been described for infectious bronchitis disease, *Staphylococcus sp., Ornitholobacterium rhinotracheale, Mycoplasma gallisepticum*, and *Escherichia coli* (45). However, no evidence was found for such co-infections during diagnostics. Secondly, non-pathogenic stress factors, such as dietary calcium stress, could have caused more severe clinical signs of the AIV infection in the field (46). Thirdly, the difference in clinical signs may be caused by the different ages of the infected hens: the hens in the field were 82 weeks old, while the hens in the experiment were only 6 weeks old. It has previously been demonstrated that non-reproductive hens shed less LPAIV than reproductive hens (47). Moreover, it has been shown that non-reproductive hens infected with an H3N1 virus infection, which circulated in poultry in Belgium in 2019, showed less severe clinical signs than hens that were laying eggs (48).

Both environmental diagnostic methods showed their potential for detection of AIV in water. The H10-specific rRT-PCR proved more sensitive than the M-PCR, which is likely due to differences in the match between this virus strain and the primers and probe used in these PCRs. The biggest advantage of the 1L method is the relatively small sampling volume, which makes the sampling and filtering process manageable. Another advantage of the 1L method is that the procedure takes less time than the procedure of the 50L method. However, the 50L method makes it possible to filter the surface water directly in the field, using portable equipment. After filtering, only the filter needs to be transported to the laboratory. Moreover, it has been shown that the 50L method can also be used for filtering other viruses, such as PEDV and Arboviruses (data not shown). PEDV, which was also used as an intern control, was detectable with CTs of around 20, when 200 μl of virus with a titer of 6.3 × 10^4^ plaque-forming units/ml was added to a surface water sample of 50L. No detection limit of the 50L method was determined for AIV, but for the 1L method the detection limit was determined using environmental water samples that were spiked with LPAIV H5N7 virus. The detection limit was determined at 0.33 log_10_ egg infectious dose (EID_50_)/ml, which means that the 1L method is able to detect a minimum of 2140 virus particles in 1L of water. Looking at the mean cloacal HPAIV shedding of ducks—which is 3.8 log_10_ EID_50_/ml and takes on average 6.6 days (47)—this environmental diagnostic method may still be able to detect AIV when 9 infected ducks shed virus in a pond or waterway of 10,000L (assuming that a duck produce on average 60 ml feces per day). As discussed, AIV can remain infective for varying lengths of time in water. This was shown for several AIVs which were added to different types—fresh, brackish and salt—of natural water: for three LPAIVs the viral titer decreased with 1 log_10_ TCID_50_/ml within five to seven days (49). When the survival of two other LPAIVs was investigated, a maximum survival of 2 weeks was defined (10). Our study showed that AIV was still detectable with the environmental diagnostic methods after at least 2 weeks. During these 2 weeks, the mean ambient temperature, measured by the nearest weather station, was 12.6°C (50). Due to the relatively low detection limit and long persistence of the virus, it appears likely that in areas with several infected wild birds, the presence of AIV in water can be measured using these environmental diagnostic methods. Therefore, these methods could be useful for screening purposes of AIV in wetlands with high bird numbers, important wintering sites for migratory water birds or in free-range areas of farms. The use of environmental diagnostic methods was also shown in other studies. AIV was detected in 12 out of 597 water samples collected from the wetlands in California (14) and in 4 out of 9 water samples collected from the Dombes ponds in France during fall migration of wild birds in 2009 (13) using similar methods. Furthermore, AIV was isolated from 12 out of 102 water samples collected from different lakes in Alaska which were important breeding sites for migratory water birds (12), and from 1 out of 265 water samples collected from the Izumi plain, an overwintering site of endangered cranes and of many other migratory birds (15).

To conclude, this study showed the potential of two methods to detect AIV in water during field infections. Detecting AIV in the environment using water samples may be a good tool to improve AIV surveillance and may assist in deciding to lift precautionary measures in outbreak situations.

## Data Availability Statement

All datasets analyzed for this study are included in the article.

## Ethics Statement

The animal study was reviewed and approved by the Centrale Commissie Dierproeven (CCD), Den Haag, Netherlands.

## Author Contributions

EG, AE, WP, and NB: conceptualization. EG, NDB, RH, RHH, and WV: methodology. RH, RHH, WP, and WV: validation. EG: data curation. AE, WP, and NB: supervision. SB and ME: phylogenetic analysis. EG and NDB: writing—original draft preparation. EG, AE, NDB, RH, WV, SB, ME, RHH, WP, and NB: writing—review and editing.

## Conflict of Interest

NDB was employed by the company GD-Animal Health, Deventer, Netherlands. The remaining authors declare that the research was conducted in the absence of any commercial or financial relationships that could be construed as a potential conflict of interest.

## Supplementary Material

Figure S1 Maximum likelihood phylogenetic tree based on the nucleotide sequence of the HA segments from influenza strains related to A/Chicken/Netherlands/17013178-006-010/2017. The evolutionary history was inferred by using RaxML (39) and utilizing the maximum likelihood method based on the General Time Reversible (GTR) model with a gamma-distributed variation of rates and 1000 bootstrap replicates. Bootstrap support values are indicated at the corresponding branch. The optimal phylogenetic tree is shown, and is drawn to scale. The GISAID accession numbers of the viruses are shown in the trees. The H10N7 viruses isolated from the hens and from the water sample are marked in red.

## Abbreviations

AIV: avian influenza virus
EID_50_: egg infectious dose
HA: haemagglutinin
H&E: hematoxylin and eosin
HI: Haemagglutination Inhibition
HPAIV: highly pathogenic avian influenza virus
IHC: immunohistochemistry
IVPI: intravenous pathogenicity index
LPAIV: low pathogenic avian influenza virus
M-PCR: rRT-PCR targeting the Influenza M gene
NA: not applicable
NGS: next-generation-sequencing
PEDV: Porcine Epidemic Diarrhea virus
rRT-PCR: Real-time Reverse Transcriptase Polymerase Chain Reaction
RT: room temperature.
