# Supplementary figures and images for: Detection of Low Pathogenic Avian Influenza Virus Subtype H10N7 in Poultry and Environmental Water Samples During a Clinical Outbreak in Commercial Free-Range Layers, Netherlands 2017
Source: Front Vet Sci. 2020 May 5;7:237. doi: 10.3389/fvets.2020.00237 (PMC7232570; doi:10.3389/fvets.2020.00237)

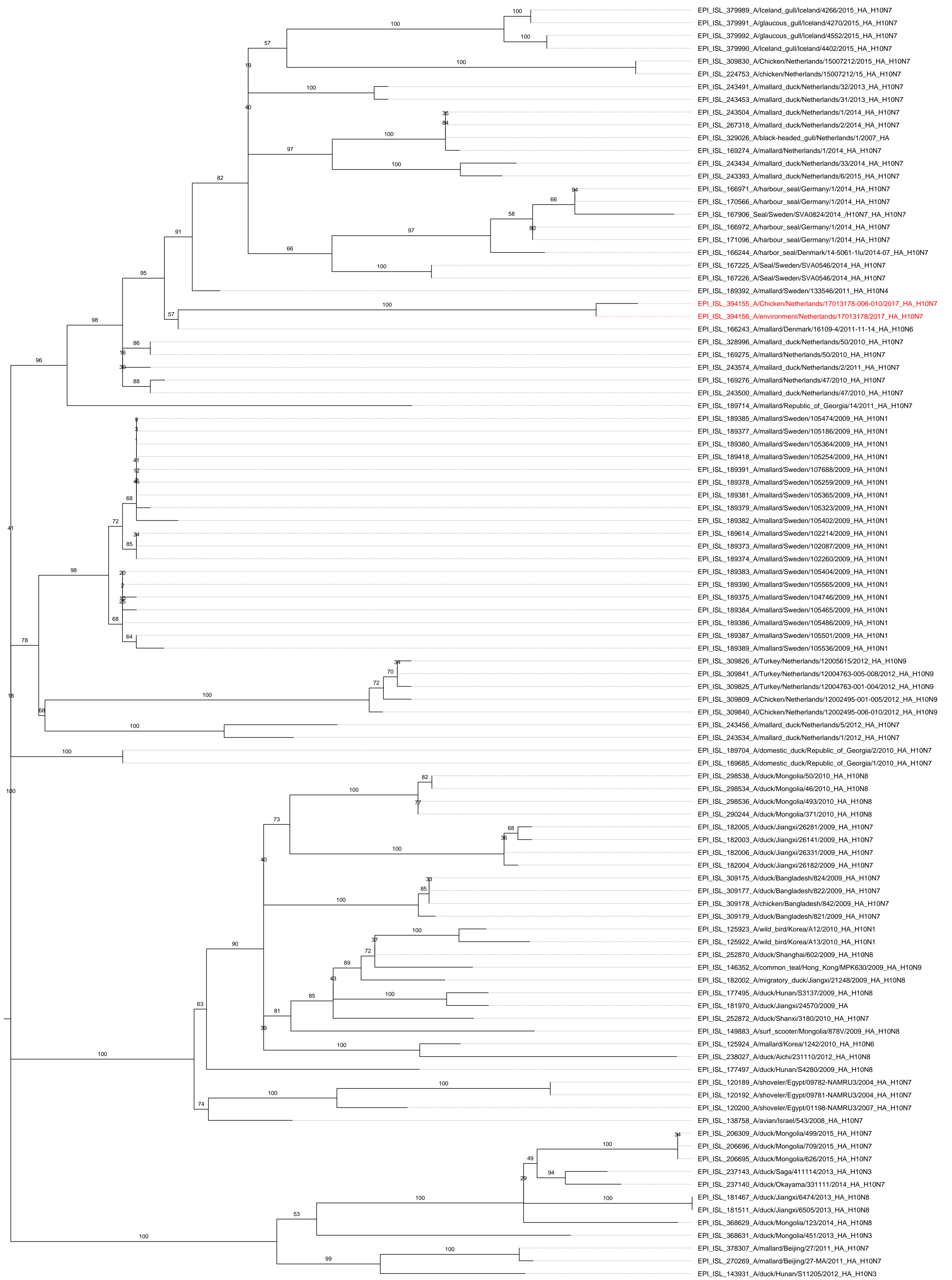

Supplement: Figure S1 — Maximum likelihood phylogenetic tree based on the nucleotide sequence of the HA segments from influenza strains related to A/Chicken/Netherlands/17013178-006-010/2017. The evolutionary history was inferred by using RaxML (39) and utilizing the maximum likelihood method based on the General Time Reversible (GTR) model with a gamma-distributed variation of rates and 1000 bootstrap replicates. Bootstrap support values are indicated at the corresponding branch. The optimal phylogenetic tree is shown, and is drawn to scale. The GISAID accession numbers of the viruses are shown in the trees. The H10N7 viruses isolated from the hens and from the water sample are marked in red. [file Data_Sheet_1.PDF]
